# Improving the accuracy of infrared measurements of skin temperature

**DOI:** 10.1186/2046-7648-4-S1-A140

**Published:** 2015-09-14

**Authors:** Allen Curran, Mark Klein, Mark Hepokoski, Corey Packard

**Affiliations:** 1 ThermoAnalytics, Inc., Calumet, MI, USA

## Introduction

In principle, infrared (IR) imagery of exposed skin and clothing can provide a valuable source of data for thermo-physiological studies. In practice, and despite the fall in the cost of IR cameras, infrared imagery is not universally collected during human subject testing. One reason for this may be the relatively poor accuracy of IR cameras (typically ±2 °C). The repeatability of the measurements taken with a particular IR camera (both spatially and temporally) is much better than its accuracy, i.e., close to the camera's sensitivity. An IR camera's sensitivity (and repeatability) is typically on the order of hundredths of a degree Celsius. Consequently, a reference with known temperature and emissivity placed in the camera's field-of-view when taking measurements can provide two potential benefits: First, measurement error can be reduced by using a calibration procedure. Second, IR imagery taken by different IR cameras during different test episodes can be directly compared.

## Methods

Two IR cameras (a FLIR E60 and a FLIR ONE) were used to collect IR imagery during a simple human subject test. An IR thermometer (Omega OSXL450) was also used to make spot temperature measurements. Calibration curves (that map instrument readings to reference temperatures) were created for the IR cameras and the thermometer using a hot/cold blackbody calibration source (Omega BB701). The test subjects were asked to sit outside without jackets for 10 minutes where the ambient temperature was 4 °C. The subjects were subsequently brought indoors where the ambient temperature was approximately 20 °C. Forehead skin temperature measurements were made using the IR cameras and the IR thermometer. The hot/cold blackbody and a second heated-only blackbody (Omega BB702) were placed in the field-of-view of the cameras to provide reference temperatures that could be used to correct the measured forehead temperature through interpolation. Calibration procedures were evaluated by examining the bias [1] between the measurements made of the same forehead skin temperature.

## Results

Measured forehead temperatures for a single individual are shown below, before and after correction using the calibration curve and the in-scene blackbodies.

## Discussion

The calibration procedure did not appear to positively impact measurements made with the FLIR ONE. Calibration did improve the bias between measurements made with the FLIR E60 and the Omega OSXL 450 from 1.0 °C (uncorrected) to 0.58 °C (corrected with the calibration curve) to 0.48 °C (corrected with the in-scene blackbodies).

## Conclusion

Both calibration procedures improved the agreement between measurements made with the FLIR E60 and the Omega OSXL450. Placing blackbodies in the camera's field of view has the advantage of ensuring that the calibration information is always available and current. The FLIR ONE, which is a low-cost IR camera marketed to consumers, does not appear suitable for collecting absolute temperature data even when a calibration procedure is employed.

**Figure 1:**
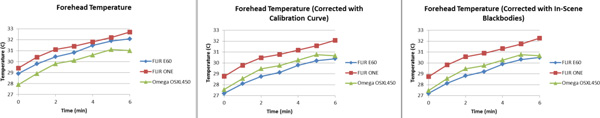


## References

[B1] Psikuta Agnes Validation of the Fiala multi-node thermophysiological model for UTCI application International Journal of Biometeorology 2012 56 3 443 460 May 10.1007/s00484-011-0450-5 21656016

